# Traditional bonesetters in northern Ghana: opportunities for engagement with the formal health sector

**DOI:** 10.11604/pamj.2020.37.248.22420

**Published:** 2020-11-18

**Authors:** Tolgou Yempabe, Anthony Edusei, Peter Donkor, Alexis Buunaaim, Charles Mock

**Affiliations:** 1 Department of Surgery, Tamale Teaching Hospital, Box 16, Tamale, Ghana,; 2 Kwame Nkrumah University of Science and Technology, School of Public Health, Kumasi, Ghana,; 3 Department of Surgery, Kwame Nkrumah University of Science and Technology, Box 1939, Kumasi, Ghana,; 4 Department of Surgery, University for Development Studies, Tamale, Ghana,; 5 University of Washington, Box 359960, Harborview Medical Center, 325 Ninth Avenue, Seattle, WA 98104, USA

**Keywords:** Injury, traditional bonesetter, Ghana, Africa

## Abstract

**Introduction:**

we sought to explore the knowledge and practices among traditional bonesetters (TBSs) in the Northern Region of Ghana and to assess opportunities for their engagement with the formal health sector.

**Methods:**

we identified 28 TBSs widely distributed in the Northern Region. They were interviewed using qualitative and quantitative methods, regarding their background, training, current practices, opinions regarding orthodox care, and interests in future linkages with the formal health sector.

**Results:**

most TBSs (67.9%) had no formal education and most (85.7%) learned their skills from older family members. Their treatments included reasonable versions of closed reduction and immobilization, but also use of locally-derived concoctions and spiritual aspects, such as incantations. Only 21.4% regularly referred complications to hospital. Nonetheless, all endorsed advantages to orthodox care, such as X-rays (100%), record keeping (100%), and pain management (85.7%). Almost all (96.4%) expressed an interest in training courses or other engagement with the formal health sector. Topics in which they were interested for training included record keeping (100%), pain management (85.7%), and management of open fractures and complications (82.1%).

**Conclusion:**

factors making linkage between TBSs and the formal health sector difficult included low levels of formal education, training through secretive in-family methods, and spiritual and mystical aspects of their practice that might make communications about modern medicine difficult. Nonetheless, most indicated interest in linking with modern care, especially through training courses. Topics they suggested for such courses provide a foundation to build on in future efforts to engage TBSs with the formal health sector.

## Introduction

Musculoskeletal injuries are a major public health problem and cause a large burden of disability and suffering globally. From 1990 to 2010, the percentage of all disability adjusted life years (DALYs) lost due to musculoskeletal disorders increased from 4.7% to 6.8%. They are ranked as the second largest contributor to disability globally [1]. Musculoskeletal injuries often result from diverse causes; however, in Ghana road traffic crashes and other accidents remain the most common causes [2,3]. These injuries require immediate and appropriate care to avoid complications and to assure good functional outcome. In a developing country such as Ghana, there are generally shortages of orthopaedic surgeons [4]. In 2014, there were 24 orthopaedic surgeons in Ghana compared to 23,956 in the United States [5]. This equates to 0.9 orthopaedic surgeons per million population for Ghana compared to 75.2 per million in the USA. Nonetheless even in places where orthopaedic services are available in the formal health system, many people still utilize the services of traditional bonesetters (TBS). In Nigeria, 85% of patients with fractures reported having used the services of TBSs [6,7]. In Ghana, one hospital reported that 63% of fracture patients who presented left to seek the services of TBSs [4]. Nonetheless, complications such as nonunion, infection, and gangrene are often reported after TBS treatment [6-12]. With the undeniable high patronage of TBSs in most African settings, there have been several calls for integrating TBSs to the formal health sector [7,13]. However, potential for this linkage has been only minimally addressed. The present study sought to explore the knowledge and practices among TBSs in the Northern Region of Ghana and to assess the opportunities for engagement with the formal health sector.

## Methods

Ghana is a lower-middle income country in West Africa with a population of 28 million people [14]. The study was conducted in the Northern Region of Ghana using a mixed method that combined both qualitative and quantitative approaches. A total of 28 TBSs were interviewed. Ten of these were contacted with the aid of a list from the northern branch of “Ghana Federation of Traditional Medicine” (GHAFTRAM). The remaining TBSs were selected through snowball sampling starting with the local head of GHAFTRAM. Snowball sampling is a method often used when there are limited or no lists of people whom the researcher is seeking to interview. Once a few suitable people are identified, in addition to being interviewed, they are also asked about whether they know other potential respondents who would qualify for the study. Hence, the number of potential respondents grows by such contacts as the study proceeds, in the same way in which the size of a snowball grows as it rolls. Twenty-nine (29) TBSs were identified and invited to participate. One declined and 28 were interviewed. These 28 TBSs were geographically dispersed across the Northern Region, being located in 16 towns or villages.

An open-closed questionnaire was used to collect quantitative data on the socio-demographic characteristics and knowledge of TBSs on the various forms of bone injury while a semi-structured interview provided in-depth views of practices of TBSs. The knowledge and practices of these practitioners were assessed regarding how they become professional TBSs, the types of bone injuries they know and the steps they follow in the treatment of fractures. The problems they encounter and how they solve them. whether they refer some of the patients to the hospital, perceived advantages of TBS over orthodox care or vice versa, would they be interested if the Ministry of Health (or other group in orthodox medicine) were to develop training courses for TBSs, what topics would they like to be included in such courses, and their suggestions on how collaboration between orthopedic surgeons and TBS can be made. Interviews were primarily 19 (67.9%) conducted in the local languages through interpreters, with 9 (32.1%) interviews being conducted in English. Data collection and analysis were intertwined and interactive. Stata version 14.0 was used for the analysis of the quantitative data. Content analysis of the qualitative data was done manually after interview results have been transcribed. Each interview was scrutinized carefully to identify main categories and leading concepts. Themes and emerging ideas were explored in subsequent interviews. In the results below, quantitative data are presented when available, supplemented by themes that emerged from the interviews. The Committee for Human Research and Publication Ethics of the Kwame Nkrumah University of Science and Technology approved this study. Written consent was obtained from participants. To ensure anonymity and confidentiality, codes were used for each participant.

## Results

**Socio-demographic characteristics of traditional bonesetters:** the 28 TBSs involved in this study were all males, their ages ranged from 19 to 75 years, and they came from multiple ethnic backgrounds (Table 1). All of them combined their bone setting practices with farming activity. Most had no formal education.

**Table 1:** socio-demographic characteristics of traditional bonesetters

Variables	Responses	Frequency n=28	Percentage 100%
**Gender**	Male	28	100
	Female	0	0
**Age**	19-39	3	10.8
	40-59	16	57.1
	60 and above	9	32.1
**Religion**	Christianity	2	7.1
	Islam	25	89.3
	Traditional believer	1	3.6
**Ethnicity**	Dagombas	19	67.8
	Mamprusis	4	14.3
	Gonjas	3	10.7
	Busangas	1	3.6
	Dagaatis	1	3.6
**Education Qualification**	No formal education	19	67.9
	Primary/Junior high school	6	21.4
	Senior high school	0	0
	Post-secondary	2	7.1
	Tertiary	1	3.6

**Traditional bonesetters´ training and knowledge:** most TBSs 24(87.5%) reported that their practice was family based, handed down from one generation to another. A chosen family member should have certain personal characteristics including honesty, responsibility, ability to keep family secrets, and be empathetic. He will take over the practices after the death of the father hence the duration of training is not specified even though the majority of TBSs quoted 15 years and above. Other routes to becoming a TBS included an apprenticeship (3, 10.7%) and divine calling (1, 3.6%). All of 28 TBSs agreed that a bonesetter has to have knowledge of natural remedies for healing as well as the ability to manipulate the body with the hands. The majority of the bonesetters were able to describe different types of bone injury. They knew different bones and their numbers especially the upper limb and lower limb as shown in Table 2. They were less familiar with injuries to the hand, foot, spine, pelvis, or skull. They diagnosed fractures and dislocations based on the cardinal signs of pain, swelling, deformity, abnormal movement, crepitus, loss of function and presence of a gap on the affected site.

**Table 2:** knowledge of types of bone injuries

Type of injury	Frequency	Percentage
Upper limb injury(excluding hand)	28	100%
Lower limb injury(excluding foot)	28	100%
Hand injury	22	78.5%
Spine & Pelvic injury	21	75%
Joint injury	20	71.4%
Foot injury	20	71.4%
Skull injury	14	50%

**Treatment practices:** all the TBS use conventional medical approaches to manage fractures. They start by taking a history to ascertain the cause of injury, followed by palpation and feeling the affected part with their fingers to identify a gap and number of fragments to ascertain the nature and severity of the fracture. Some of them said the availability of an x-ray makes the diagnosis easier as it can ascertain the nature and anatomical site of fracture. One indicated that he preferred the patient to go to the hospital for first aid and x-ray before coming to him for treatment. After the diagnosis is ascertained, they pull the affected part, with the aid of an assistant, to reduce the fracture, followed by massage and palpation. Five (17.9%) use incantations during the process because they believe that healing power is from their ancestors and gods. Hot water was used by 5 (17.9%) to dissolve clots so that healing can take place. Once they are sure about the reduction of the fracture, a locally made concoction, usually termed “black medicine”, is applied to aid in the healing process. The composition of this medicine differs from one TBS to another but typically uses leaves, tree bark, roots, or other plant material. Shea butter and other concoctions are used by some TBSs. The specific composition of the medicines is usually a family secret. Two (7.1%) applied black medicine after scarification to accelerate the penetration of medicine to the fracture site. To maintain the reduction of fracture, cotton wool or bandage is applied first to avoid injury to skin, followed by application of a mat, bamboo board, or wood, which is then tied. A cloth sling is fixed around the neck if it is an upper limb fracture. None of them reported using leaves for bandaging.

Patients are reviewed every three days and bandages/splints are released to apply the black medicine or other concoctions. This process is continued until the fracture heals. The criteria used to define a healed fracture were the absence of a gap between the broken fragments, minimal deformity, and the return of painless function of the affected part. The TBSs involved in the study had a reasonable idea of factors affecting fracture healing. According to them, old age is one of the conditions that affect fracture healing because of the low vitality and less regenerative power. They recognized that in young age groups, the healing of a fracture takes one months or less whereas it takes about two months in middle age, and three months in old age. To aid in ascertaining when the fracture is healed, 4 (14.3%) of the TBSs asked the families to supply a chicken. The TBS would then break either the wing or leg of the chicken, depending on whether their patient had a lower or upper extremity fracture. The chicken is observed and the day it starts walking they will ask the patient to walk because they believe healing has taken place.

**Knowledge of complications and their management:** TBSs recognize swelling as part of healing process and identify complications when they notice increasing swelling associated with blisters, maceration, change of skin color, foul smelling discharge, and fever. All indicated that they periodically encounter problems with infection during treatment. Despite recognizing these complications, only a minority regularly refers patients with infections to the hospital. Most will use a variety of treatments, including locally derived concoctions or antibiotics obtained from nurses at clinics in their areas (Table 3). TBSs typically recognize the difficulty in dealing with some fractures especially those with soft tissue interposition and those in specific bones, including the patella, clavicle, chest, pelvis, spine, and skull. All agreed that the most difficult fracture to treat is a displaced patella. The majority 23 (82.1%) recognized the difficulty of treating open fractures and prefers to refer to hospital for wound management before the clients come back for fracture treatment. A minority said they do not refer and treat all kinds of open fractures, using a variety of means including Shea butter, antibiotics, or locally derived concoctions.

**Table 3:** management of infection

Treatment modalities	Frequency	Percentage
Refer to hospital	6	21.4%
Treat with antibiotics	7	25%
Treat with “black medicine”	8	28.6%
Treat with other concoction	7	25%
Total	28	100%

**Perceived advantages of traditional bonesetters vs. orthodox care:** most TBSs felt that the care that they provided had advantages over orthodox care. The main reasons advanced were cheaper fees, easy accessibility, and quicker healing (Table 4). Cultural beliefs and ability to treat both physical and spiritual aspects also played a role. All the bonesetters agreed that the orthodox care also has some advantages over TBS care. The main reason advanced were availability of X-rays, use of record keeping, and proper pain management (Table 5).

**Table 4:** perceived advantages of traditional bonesetters over orthodox care

Perceived advantages	Frequency	Percentage
Cheaper fees	21	75%
Easy accessibility	20	71.4%
Quick healing	20	71.4%
Cultural beliefs	18	64.3%
Quick service	16	57.1%
Treat both physical and spiritual	11	39.3%
No amputation	10	35.7%
Use of incantations	8	28.6%
No operation	8	28.6%
No persistent pain after treatment	6	21.4%

**Table 5:** perceived advantages of orthodox care over traditional bonesetters

Perceived advantages	Frequency	Percentage
X-ray facility	28	100%
Record keeping	28	100%
Proper pain management	24	85.7%
Adequate wards to admit patients	17	60.7%
Resuscitation	13	46.4%

**Interests in engagement with the formal health sector:** almost all (96.4%) of the TBSs expressed an interest in engagement with the formal health sector. They were especially interested in exchange of contacts (100%), regular meetings (96.4%), including training course (96.4%). Among the topics that they would like to have included, the most common were record keeping, pain management, and management of open fractures and complications (Table 6). Although many TBSs were illiterate, they indicated that the record keeping could be done by literate family members or others in the community.

**Table 6:** topics of interest for traditional bonesetter training

Topics of interest	Frequency	Percentage
Record keeping	28	100%
Pain management	24	85.7
Open fractures and wounds management	23	82.1
Complications of fractures	23	82.1
Bone healing	17	60.7
Infection management	12	42.9
Tendon injury	5	17.9

## Discussion

In this study, we sought to explore the knowledge and practices among TBSs in the Northern Region of Ghana and to assess the opportunities for engagement with the formal health sector. We found that most TBSs have no formal education of any kind (literacy or medical training) and are trained primarily by older family members, with TBS skills passed from generation to generation. Many of the treatments appear to be reasonable versions of closed reduction and immobilization. There is also extensive use of locally derived concoctions and spiritual aspects, such as incantations. Most TBSs understood signs of infection, but only a minority regularly referred complications to the hospital. Nonetheless, all TBSs indicated that there were advantages to orthodox care and almost all expressed an interest in training courses or other engagement with the formal health sector. They also indicated a reasonable set of topics in which they were interested in obtaining training. In Ghana, as in most Africans countries, there are both traditional and orthodox health care systems coexisting side by side. Earlier studies found that people use the services of TBSs even in places where modern orthodox care is nearby [7,10]. For example, Aries et al found that most (63%) fracture patients who presented to a Ghanaian hospital left to seek the services of TBSs [8]. Similarly, in Nigeria, up to 85% of patients with fractures have been reported to use the services of TBSs [7].

Practices of TBSs in other countries have been identified as being similar to those reported in this study. For example, Dada *et al*. reviewed 31 publications about TBS practices in Nigeria and other African countries [9]. They identified use of similar closed manipulation and immobilization techniques, as well as use of local concoctions and incantations and other spiritual aspects. Likewise, these studies showed similar training methods of generation-to-generation transmission of knowledge, with emphasis on keeping family secrets. These factors lead to lack of use of scientific information and no role of peer review [9]. Multiple publications have noted problems arising from TBS care, such as malunion, nonunion, infections, gangrene, and related limb loss. In light of these problems, there have been calls for discouragement of TBS practice. Accomplishing this would be difficult, however, given the high rates of patronage, especially in rural areas with limited availability of orthodox care [6,8,9,11,12]. An alternative to discouraging use of TBSs is to link them with the formal health care system, such as through providing them with training to recognize their limitations. This has been successfully tried in at least two African countries. In Ethiopia, a one-day course on safe fracture care was given to 112 TBSs. This resulted in a notable decrease in amputations arising from the TBSs practice, with 49 such amputations in the two years before the course, down to 25 in the two years afterwards [10]. In Nigeria, a two-year prospective study noted a statistically significant difference between the rate of gangrenous limbs, infection, and non-union and mal-union in tibial shaft fractures at a TBS center at which a one-day course was conducted compared with an untrained center [15].

These small studies in Africa are backed up by similar efforts elsewhere. In China, Chinese-style doctors are trained in care of diseases including pain control, fracture and sprain management. The practice is regulated and practitioners undergo structured training resulting in minimal complications [11]. In Turkey, traditional practitioners have been trained to refer difficult cases [9]. In Nepal, training programs for rural village health workers increased knowledge base and working skills, which persisted for 6 years after the trainings [16]. Similar training efforts have not been reported from Ghana. An older report from the 1980s indicated that efforts at formal cooperation between medical doctors and TBSs had been tried in Northwest Ghana, but without published evaluations [17]. There are reports of efforts to link other types of traditional healers with the formal health sector, with disappointing results. In 1979, the Primary Health Training for Indigenous Healers Project (PRHETIH) was started in the northwest Ghana [18]. Ten years later, the program´s impact was evaluated and found to have only minimally influenced the healer´s treatment methods. The program´s failure could be explained, in part, by the traditional healer´s mystical and spiritual explanations for disease causation [13]. Any future efforts at training of TBSs in Ghana could reasonably rely on the list of topics that TBSs in this study suggested (Table 6). Other priorities for such training would include hygiene and infection control, recognition of the limits of their practice, and the need to refer difficult cases such as open fractures and infections. Interestingly, the most commonly requested topic for training was record keeping. This is also the factor that all TBSs identified as an advantage of orthodox care over TBS care (Table 5). Establishing some element of record keeping for TBSs would enable future efforts to better monitor and regulate the care they provide. In terms of training methods, the fact that most TBSs are illiterate would indicate a need to rely on demonstrations and illustrations rather than written material. The TBSs´ belief system in which spiritual and mystical factors are prominent will need to be carefully considered and addressed for any training to be successful.

Before drawing conclusions from the data, the limitations of the study must be addressed. First, TBSs were chosen for interviews based on the snowball method of identifying contacts. It would have been preferable to use a random sample drawn from a list of TBSs. However, there is no such list available and it is not known how many TBSs there are. Hence, the snowball method was the best available option. Moreover, most prior studies that have reported interviews with TBSs have usually had sample sizes in the range of only 2 - 8 TBSs interviewed [6-8,19], making the current study one of the largest. Second, the need for interpreters may have led to loss of detail. Third, a variety of locally derived concoctions were used, but due to the emphasis on family secrecy, it was not possible to learn further details on these. Finally, all of the data were gathered from self-report of the TBSs and there is no way to validate the information they provided. Despite these limitations, the study offers the advantages of having mixed methods data gathering on a sizeable sample of TBSs, who were from multiple ethnic backgrounds and were widely distributed across the Northern Region.

## Conclusion

TBSs in the Northern Region of Ghana have several factors that would tend to make linkage with the formal health sector difficult. Most have no formal education and thus low literacy skills. Most are trained through secretive in-family methods and much of their practice is based on spiritual and mystical aspects that would tend to make communications about modern medicine difficult. They also perceive a competition with modern medicine, noting multiple benefits of their own practices in comparison to modern care. Nonetheless, all did note some benefits of modern care and almost all indicated an interest in linking with modern care, especially through training courses. They indicated a reasonable set of topics that such courses could cover. These topics provide a foundation to build on in future efforts to engage the TBSs with the modern health care system in Ghana and other similar countries globally.

### What is known about this topic

- Traditional bonesetters (TBS) are highly patronized in Africa;
- There have been calls to integrate them into the modern health care system.

### What this study adds

- The secret nature of training and the fact that mystical / spiritual powers used in the treatment protocol of TBS are limiting factors to collaborative or integrative efforts between TBS and orthodox practitioners have been recognized by various authors before now as has also been alluded to in this study;
- All TBSs noted some benefits of modern care and most indicated an interest in linking with modern care, especially through training courses;
- Topics suggested by TBSs for courses provide a foundation to build on in future efforts to engage them with the formal sector.

## References

[1] Lyn M, Peter B, Damien H, Rachelle B, Marita C, Emma S (2013). Global and Country Specific Burden of Musculoskeletal Disorders: A Report from The Global Burden of Diseases Musculoskeletal Expert Group. American College of Rheumatology 2013. Annual Meeting.

[2] Moises K, Wurapa F, Nonvignon J, Norman I, Awoonor-Williams JK, Aikins M (2011). Economic burden of motorcycle accidents in Northern Ghana. Ghana Med J.

[3] Kuubiere Callistus, Abass F (2015). Patients preference for traditional bone setters in northern Ghana. Al Ameen J Med Sci.

[4] Mock Charles, Cherian MN (2008). The global burden of musculoskeletal injuries: challenges and solutions. Clin Orthop Relat Res.

[5] Brouillette MA, Kaiser SP, Konadu P, Kumah-Ametepey RA, Aidoo AJ, Coughlin RC (2014). Orthopedic surgery in the developing world: workforce and operative volumes in Ghana compared to those in the United States. World J Surg.

[6] Nwachukwu BU, Okwesili IC, Harris MB, Katz JN (2011). Traditional bonesetters and contemporary orthopaedic fracture care in a developing nation: historical aspects, contemporary status and future directions. Open Orthop J.

[7] Omololu AB, Ogunlade SO, Gopaldasani VK (2008). The practice of traditional bonesetting: training algorithm. Clin Orthop Relat Res.

[8] Aries MJ, Joosten H, Wegdam HH, van der Geest S (2007). Fracture treatment by bonesetters in central Ghana: patients explain their choices and experiences. Trop Med Int Health.

[9] Dada AA, Yinusa W, Giwa SO (2011). Review of the practice of traditional bone setting in Nigeria. Afr Health Sci.

[10] Eshete M (2005). The prevention of traditional bone setter's gangrene. J Bone Joint Surg Br.

[11] Garba ES, Deshi PJ (1998). Traditional bone setting: a risk factor in limb amputation. East Afr Med J.

[12] Musa AA (1998). The causes of amputations in Sokoto, Nigeria. Trop Doct.

[13] Ventevogel Peter (1996). Whiteman's things: Training and detraining healers in Ghana.

[14] World Bank Group (2019). World Development Indicators.

[15] Onuminya JE (2006). Performance of a trained traditional bonesetter in primary fracture care. S Afr Med J.

[16] Shah RK, Thapa VK, Jones DH, Jones R (2003). Improving primary orthopaedic and trauma care in Nepal. Educ Health (Abingdon).

[17] Van der Horst J (1985). The role of traditional medicine in the primary health care process. An example from Upper-West Ghana. Thesis Master of Public Health.

[18] Warren DM, Bova GS, Tregoning MA, Kliewer M (1982). Ghanaian national policy toward indigenous healers. The case of the primary health training for indigenous healers (PRHETIH) program. Soc Sci Med.

[19] Edusei AK, Owusu-Ansah FE, Dogbe JA, Morgan J, Sarpong K (2015). Perspectives in musculoskeletal injury management by traditional bone setters in Ashanti, Ghana. Afr J Disabil.

